# Supplementary material for: The Systems Biology Research Tool: evolvable open-source software
Source: BMC Syst Biol. 2008 Jun 29;2:55. doi: 10.1186/1752-0509-2-55 (PMC2446383; doi:10.1186/1752-0509-2-55)
Supplement: Additional file 1 — SBRT Archive. An archive of the current version of the Systems Biology Research Tool. [file 1752-0509-2-55-S1.zip › sbrt-1.4.0/doc/developers_guide/api/sbrt/shell/mng/fba/RandomFluxVectorManager.html]

RandomFluxVectorManager


|  |  |  |
| --- | --- | --- |
| **Overview** \| **Package** \| **Class** \| **Use** \| **Tree** \| **Deprecated** \| **Index** \| **Help** | |  |
| **PREV CLASS**   **NEXT CLASS** | **FRAMES**    **NO FRAMES**     **All Classes** |  |
| SUMMARY: NESTED \| FIELD \| CONSTR \| METHOD | DETAIL: FIELD \| CONSTR \| METHOD |  |


---


## sbrt.shell.mng.fba Class RandomFluxVectorManager

```
java.lang.Object
  sbrt.shell.mng.AbstractProcessManager
      sbrt.shell.mng.fba.FbaManager
          sbrt.shell.mng.fba.RandomFluxVectorManager
```

**All Implemented Interfaces:**: KernelProcessManager<java.lang.String,java.lang.String>, ProcessManager<java.lang.String,java.lang.String>

**Direct Known Subclasses:**: CdHarFluxVectorManager

---

``` public abstract class RandomFluxVectorManager extends FbaManager ```

This abstract class provides a foundation for random flux vector
managers. It contains a useful keyword as well as a set of utility functions.

**Author:**
:   This class was written and documented by Jeremiah Wright while
    in the Wagner lab.

---

| **Field Summary** | |
| --- | --- |
| `static java.lang.String` | `ALGORITHM`             The keyword used to denote a random flux vector algorithm. |

**Fields inherited from class sbrt.shell.mng.fba.FbaManager**

`CONSTRAINT_TOLERANCE, DEFAULT_CONSTRAINT_TOLERANCE, FLUX_VECTOR_FILE, FLUX_VECTOR_FILE_NAME, optionalKeywords, requiredKeywords, RXN_CATALYST_FILE, RXN_FILE, SYSTEM_SOLUTION_FILE`

**Fields inherited from class sbrt.shell.mng.AbstractProcessManager**

`COMPLETED, DATA_HEADERS, DEV_NULL, ELAPSED_TIME, FILE_FORMAT, INPUT_FILE, INPUT_FILE_FORMAT, INPUT_FILE_NAME_FILE, ITERATIONS, OUTPUT_FILE_FORMAT, OUTPUT_FILE_NAME, OUTPUT_FILE_NAME_FILE, PERCENT, PERCENT_COMPLETED, SEED, ZERO_CUTOFF`


**Constructor Summary**

`RandomFluxVectorManager()`


| **Method Summary** | |
| --- | --- |
| `static FluxConstraints` | `getConstraints(ManagerInput<java.lang.String,java.lang.String> input, java.io.PrintWriter out, Fluxome fluxome)`             Opens the file denoted by `FbaOptManager.CONSTRAINTS_FILE` in the provided manager input and returns the set of flux constraints it contains. |
| `static FluxConstraints` | `getConstraints(java.lang.String fileName, Fluxome fluxome)`             Returns the set of constraints contained in the file with the provided name. |
| `static IrrevRxnFluxome` | `getFluxome(ManagerInput<java.lang.String,java.lang.String> input, java.io.PrintWriter out)`             Creates and returns a fluxome based on the provided input. |
| `static java.util.Map<java.lang.String,java.lang.Double>` | `getFluxVector(ManagerInput<java.lang.String,java.lang.String> input, java.io.PrintWriter out, Fluxome fluxome, double tolerance)`             Opens the file denoted by `FbaManager.FLUX_VECTOR_FILE` in the provided manager input and returns the flux vector it contains. |
| `static java.util.Map<java.lang.String,java.lang.Double>` | `getFluxVector(java.lang.String fileName, Fluxome fluxome, double tolerance)`             Returns the flux vector contained in the file with the provided name. |

**Methods inherited from class sbrt.shell.mng.fba.FbaManager**

`getCatalysts, getCatalysts, getConstraintTolerance, getConstraintTolerance, getFluxome, getFluxome, getFluxome, getFluxome, getFluxomeSolution, getMultiFluxVectorOutputFile, getMultiFluxVectorOutputFile, getRxnNameExprFormat, getRxnNameHeaders, getRxnNameHeaders, getSingleFluxVectorOutputFile, getSingleFluxVectorOutputFile, printFluxomeInfo, writeFluxome, writeFluxome`

**Methods inherited from class sbrt.shell.mng.AbstractProcessManager**

`check, closeFile, closeRm, execute, getFileFormat, getFileFormat, getFileNameSource, getIterations, getSeed, getTextFileFormat, getTextFileFormat, getZeroCutoff, getZeroCutoff, parseInt, parseLong, printProgress, printProgress, printProgress, runProcess, setInput`

**Methods inherited from class java.lang.Object**

`clone, equals, finalize, getClass, hashCode, notify, notifyAll, toString, wait, wait, wait`

**Methods inherited from interface sbrt.shell.mng.KernelProcessManager**

`getProcess`

**Methods inherited from interface sbrt.shell.mng.ProcessManager**

`check, execute, execute, getOptionalKeys, getRequiredKeys, setInput, setInput`

**Field Detail**

### ALGORITHM

```
public static final java.lang.String ALGORITHM
```

:   The keyword used to denote a random flux vector algorithm.

    **See Also:**: Constant Field Values


**Constructor Detail**

### RandomFluxVectorManager

```
public RandomFluxVectorManager()
```


**Method Detail**

### getFluxVector

```
public static java.util.Map<java.lang.String,java.lang.Double> getFluxVector(ManagerInput<java.lang.String,java.lang.String> input,
                                                                             java.io.PrintWriter out,
                                                                             Fluxome fluxome,
                                                                             double tolerance)
```

:   Opens the file denoted by `FbaManager.FLUX_VECTOR_FILE` in the
    provided manager input and returns the flux vector it contains.

    :   **Parameters:**: `input` - the input containing a value for `FLUX_VECTOR_FILE`.: `out` - the print writer to which a status message will be printed.: `fluxome` - the fluxome to which the flux vector corresponds.: `tolerance` - the amount by which fluxes in the vector are allowed to violate their defined constraints. **Returns:**: the flux vector contained in the file indicated by `#FLUX_VECTOR_FILE` in the provided input. **See Also:**: `getFluxVector(String, Fluxome, double)`

---


### getFluxVector

```
public static java.util.Map<java.lang.String,java.lang.Double> getFluxVector(java.lang.String fileName,
                                                                             Fluxome fluxome,
                                                                             double tolerance)
```

:   Returns the flux vector contained in the file with the provided name.
    This file must have the format of a row-oriented map file, the lines must
    be parsable using `FluxVectorFormatV1`, and a flux must be present
    for every reaction in the provided fluxome. The flux vector must also lie
    within the flux space defined by the provided fluxome.

    :   **Parameters:**: `fileName` - the name of the file to be read.: `fluxome` - the fluxome to which the fluxes correspond.: `tolerance` - the amount by which fluxes in the vector are allowed to violate their defined constraints. **Returns:**: the flux vector contained in the file with the provided name. **Throws:**: `ApplicationException` - if the flux vector in the file does not lie within the flux space defined by the provided fluxome. **See Also:**: `RomiFile`, `Fluxome.isFluxVector(Map, double)`

---


### getConstraints

```
public static FluxConstraints getConstraints(ManagerInput<java.lang.String,java.lang.String> input,
                                             java.io.PrintWriter out,
                                             Fluxome fluxome)
```

:   Opens the file denoted by `FbaOptManager.CONSTRAINTS_FILE` in the
    provided manager input and returns the set of flux constraints
    it contains.

    :   **Parameters:**: `input` - the input containing a value for `CONSTRAINTS_FILE`.: `out` - the print writer to which a status message will be printed.: `fluxome` - the fluxome to which the constraints correspond. **Returns:**: the set of flux constraints contained in the file indicated by `#CONSTRAINTS_FILE` in the provided input. **See Also:**: `getConstraints(String, Fluxome)`

---


### getConstraints

```
public static FluxConstraints getConstraints(java.lang.String fileName,
                                             Fluxome fluxome)
```

:   Returns the set of constraints contained in the file with the provided
    name. This file must have the format of a row-oriented map file, the
    lines must be parsable using `ConstraintsFileLineFormatV1`, and
    a constraint must be present for every reaction in the provided fluxome.

    :   **Parameters:**: `fileName` - the name of the file to be read.: `fluxome` - the fluxome to which the constraints correspond. **Returns:**: the set of constraints contained in the file with the provided name. **Throws:**: `java.lang.NullPointerException` - if either argument is `null`. **See Also:**: `getConstraints(ManagerInput, PrintWriter, Fluxome)`, `FbaOptManager.getConstraints(String, Fluxome)`, `RomiFile`

---


### getFluxome

```
public static IrrevRxnFluxome getFluxome(ManagerInput<java.lang.String,java.lang.String> input,
                                         java.io.PrintWriter out)
```

:   Creates and returns a fluxome based on the provided input.
    The values for `FbaManager.RXN_FILE`,
    `FbaOptManager.CONSTRAINTS_FILE`, and
    `FbaManager.CONSTRAINT_TOLERANCE`
    are parsed and combined to create the returned fluxome.

    :   **Parameters:**: `input` - the input containing values for the listed keywords.: `out` - the print writer to which status messages will be printed. **Returns:**: a fluxome based on the provided input. **See Also:**: `FbaManager.getFluxome(ManagerInput, PrintWriter)`, `FbaManager.getConstraintTolerance(ManagerInput, PrintWriter)`, `getConstraints(ManagerInput, PrintWriter, Fluxome)`, `Fluxome.replaceConstraints(FluxConstraints, double)`


---


|  |  |  |
| --- | --- | --- |
| **Overview** \| **Package** \| **Class** \| **Use** \| **Tree** \| **Deprecated** \| **Index** \| **Help** | |  |
| **PREV CLASS**   **NEXT CLASS** | **FRAMES**    **NO FRAMES**     **All Classes** |  |
| SUMMARY: NESTED \| FIELD \| CONSTR \| METHOD | DETAIL: FIELD \| CONSTR \| METHOD |  |


---
